# Interaction of basal forebrain cholinergic neurons with the glucocorticoid system in stress regulation and cognitive impairment

**DOI:** 10.3389/fnagi.2015.00043

**Published:** 2015-04-02

**Authors:** Saswati Paul, Won Kyung Jeon, Jennifer L. Bizon, Jung-Soo Han

**Affiliations:** ^1^Department of Biological Sciences, Konkuk UniversitySeoul, South Korea; ^2^Herbal Medicine Research Division, Korea Institute of Oriental MedicineDaejeon, South Korea; ^3^Department of Neuroscience, College of Medicine, Evelyn F. and William L. McKnight Brain Institute, University of FloridaGainesville, FL, USA

**Keywords:** cholinergic neuron, stress, aging, basal forebrain, hippocampus, glucocorticoid, glucocorticoid receptor

## Abstract

A substantial number of studies on basal forebrain (BF) cholinergic neurons (BFCN) have provided compelling evidence for their role in the etiology of stress, cognitive aging, Alzheimer’s disease (AD), and other neurodegenerative diseases. BFCN project to a broad range of cortical sites and limbic structures, including the hippocampus, and are involved in stress and cognition. In particular, the hippocampus, the primary target tissue of the glucocorticoid stress hormones, is associated with cognitive function in tandem with hypothalamic-pituitary-adrenal (HPA) axis modulation. The present review summarizes glucocorticoid and HPA axis research to date in an effort to establish the manner in which stress affects the release of acetylcholine (ACh), glucocorticoids, and their receptor in the context of cognitive processes. We attempt to provide the molecular interactive link between the glucocorticoids and cholinergic system that contributes to BFCN degeneration in stress-induced acceleration of cognitive decline in aging and AD. We also discuss the importance of animal models in facilitating such studies for pharmacological use, to which could help decipher disease states and propose leads for pharmacological intervention.

## Introduction

Since its inception, the cholinergic hypothesis has generated considerable interest. It has been used to decipher the different orchestrating functions and dysfunctions in the nervous system associated with Alzheimer’s disease (AD) and other neurodegenerative diseases. This hypothesis has been used to further understand cognitive impairment and neurodegenerative diseases by evaluating and tracing brain functions in normal and aging brains (Gallagher and Colombo, 1995; Contestabile, 2011). The main components of the cholinergic pathway are: (1) the neurotransmitter acetylcholine (ACh); (2) acetylcholinesterase (AChE), which breaks down ACh; (3) choline acetyltransferase, an enzyme that synthesizes ACh; and (4) ACh receptors, specifically the nicotinic ACh receptor, and the muscarinic ACh receptor (mAChR). Evidence from previous research on normal aging (Drachman et al., 1982), AD (Whitehouse et al., 1982), and anti-cholinergic (Newhouse et al., 1988, 1994) and pro-cholinergic drug administration (Davis and Mohs, 1982) supports the major role of the cholinergic system in aged-related cognitive decline. Extensive research has established the relationship between cognitive impairment and the cholinergic system in the basal forebrain (BF; Baxter and Chiba, 1999). The involvement of the cholinergic system in regulating stress is also evident from studies that acute/inescapable stress enhanced release of ACh and induced expression of genes that regulate ACh availability in the hippocampus and prefrontal cortex (Mark et al., 1996; Kaufer et al., 1998). Cognitive processes are influenced by the acute and chronic stress-induced release of glucocorticoids, stress hormones that influence the function of the prefrontal cortex and hippocampus (Popoli et al., 2011). Stress and stress hormone plays a well-established role in mental health and impaired cognition. It is correlated with hippocampal volume and age-related cognitive decline (Lupien et al., 1994, 2009), suggesting that sustained stress, via glucocorticoid hypersecretion, leads to hippocampal damage (Uno et al., 1989). Prolonged overproduction of glucocorticoids can be detrimental to brain structure, whereas insufficient glucocorticoid signaling can lead to stress-related pathological conditions (Raison and Miller, 2003). This emphasizes the need for the careful regulation of glucocorticoid exposure. Impairment of the hypothalamic-pituitary-adrenal (HPA) axis in response to stress is also associated with cognitive dysfunction in aged animals (Issa et al., 1990; Bizon et al., 2001). In addition, HPA activity was blunt in elderly compared to young adult participants (Hatzinger et al., 2011). Therefore, new therapeutic approaches acting on the HPA axis and its receptor signaling should take into account.

Activation of the septo-hippocampal cholinergic system is considered as an important aspect in the adaptive response to stress and is influenced by neuronal and hormonal stimuli. This septo-hippocampal activation seems to initialize following activation of the pituitary-adrenocortical axis (Gilad et al., 1985; Gilad, 1987) and may then affect glucocorticoid secretion via the HPA axis (Herman et al., 1996). The HPA axis plays an important role in the adaptation to stress by modulating hippocampal activity. Thus, the hippocampus, along with cholinergic innervation from the BF, is involved in regulating the HPA axis stress response. Activation of the HPA axis mediates responses that enable an organism to maintain its homeostasis. Hence, neurodegeneration of cholinergic neurons, a pathological characteristic in AD and aging, makes the elderly vulnerable to stress, resulting in cognitive impairment.

The extent to which the cholinergic system is involved in stress, cognition, and neurological disorders has been reported in several significant research reports. Therefore, we attempt to develop a supporting background for our recent studies in rats (aged or with cholinergic lesions) on HPA axis dysfunction in response to stress and altered glucocorticoid receptor (GR) signaling in the hippocampus. We also describe the interactive link between the glucocorticoid and cholinergic systems in aging and stress.

## Overview of the Basal Forebrain Cholinergic System

**Figure 1:**
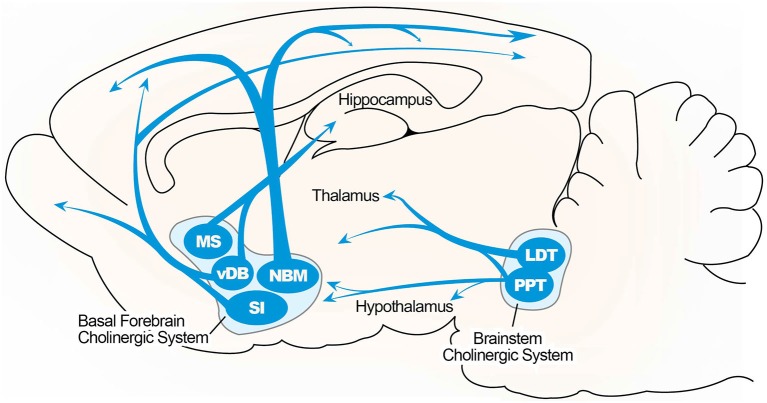
**Illustrated overview of the basal forebrain cholinergic pathway**. Cholinergic projections include the medial septum (MS), vertical limbs of the diagonal band of Broca (vDB), nucleus basalis of Meynert (NBM), and substantia innominate (SI) projecting to the hippocampus, thalamus, olfactory bulb, and cortical region. Cholinergic pontomesencephalon neurons include laterodorsal tegmental (LDT) and pedunculopontine tegmental nuclei (PPT) projecting to hindbrain, thalamus, hypothalamus, and basal forebrain.

An anatomical simplified overview of BF cholinergic neurons is summarized in Figure 1, including cells located in the medial septum (MS), the vertical limb of the diagonal band of Broca (VDB), and the nucleus basalis of Meynert–extending to the substantia innominata in the rodent brain. These structures send cholinergic projections to a broad range of neocortical sites as well as structures in the limbic system, including the hippocampus (Mesulam et al., 1983; Gallagher and Colombo, 1995). Thus, the septo-hippocampal pathway, which arises from the medial septal nucleus and nucleus of the diagonal band, is the main structure of the central cholinergic system and the main source of cholinergic innervation to the hippocampal formation. A topographical model of the septo-hippocampal pathway has been constructed based on various lesion, tracing, and immunocytochemical methods in the septal and hippocampal regions (Lewis et al., 1967; Dutar et al., 1995). The MS is connected to the hippocampus, via the fimbria and dorsal fornix, and to the medial cortex (Lewis and Shute, 1967; Teles-Grilo Ruivo and Mellor, 2013). The cornu ammonis (CA) 1 pyramidal and dentate granule (DG) cell layers in the dorsal hippocampus receive afferent inputs from the VDB, and these cell layers in the ventral hippocampus receive inputs from the both MS and VDB (McKinney et al., 1983; Nyakas et al., 1987).

### Cholinergic Hypothesis in Disease Etiology

The cholinergic hypothesis has been implicated in the etiology of AD, various types of dementia, and aging, and is rooted in degeneration of BF cholinergic neurons causing cognitive deficit (Bartus et al., 1982; Bartus, 2000; Sarter et al., 2003). This theory, however, remains controversial. Although enhancement of cholinergic function by cholinergic agents (e.g., AChE inhibitors) in AD and age-related cognitive deficit supported the hypothesis, other research subsequently pinpointed the involvement of other factors, such as dopamine projections to the frontal cortex, amyloid deposition, and increased glucocorticoid levels (Dumas and Newhouse, 2011). More specifically, hypersecretion of glucocorticoid, or A-beta-altered HPA axis function, have been implicated in hippocampal impairment in AD (Hibberd et al., 2000; Brureau et al., 2013) Additionally, elevated glucocorticoid levels and impaired GR signaling are associated with HPA dysfunction, resulting in cognitive decline in elderly subjects (Issa et al., 1990; Lupien et al., 1994; Bizon et al., 2001; Mizoguchi et al., 2009). Therefore, a possible pathophysiological link between glucocorticoids and the age-dependent decline in BF cholinergic function, especially in the CA1, CA3, and DG regions of hippocampus, has also been established (Hörtnagl et al., 1993). Craig et al. postulated a new cholinergic hypothesis version for AD, where loss of MS cholinergic input to the hippocampus induces hippocampal vulnerability, resulting in greater cognitive impairment in response to subsequent insults, such as stress or injury (Craig et al., 2011).

### Implication of Lesion Studies in Evaluating Cholinergic Innervation

Application of the cholinergic hypothesis to animal model offers the ability to evaluate functional network and molecular pathway to understand neurocognitive diseases. Selection of animal (even among rodents) is important, as they differ in cholinergic tone, receptor activation, and relevance to human basal cholinergic activity (Van der Zee and Keijser, 2011). The adoption of the rodent as an animal model may compliment studies in primates due to effectiveness and cost-efficiency. Moreover, features such as short lifespans are an important factor to be considered in the study of late-onset or aging diseases (Gallagher et al., 2011). One major pathological hallmark of neurodegenerative disease that cause cognitive decline, is the dramatic loss of BF cholinergic projection neurons with reduced cholinergic innervation to the hippocampus and neocortex (Davies and Maloney, 1976; Whitehouse et al., 1981; Arendt et al., 1983; Mesulam, 2004). Additionally, the relative loss of cholinergic neurons and the decrease of the ACh synthesizing enzyme, choline acetyltransferase in the brain of AD patients is associated with cognitive impairment (Bartus, 2000). In animal studies, neurotoxin-induced BF lesions cause similar cognitive impairments (Olton, 1990). However, lesioning cholinergic BF neurons is challenging because they are intertwined with non-cholinergic neurons and there is a risk of damaging adjacent structures.

Although BF lesions using conventional lesion methods (e.g., electrolytic) produce varying manifestations of cognitive impairment, the development of methods that selectively interrupt the BF region has been attempted (Easton et al., 2012; Baxter and Bucci, 2013). An initial approach innovatively used 192IgG-saporin, a neurotoxin with a specific affinity for cholinergic neuron cell surface receptors (Wiley et al., 1991; Baxter and Bucci, 2013). A myriad of studies picked the hippocampal and septo-hippocampal regions of the BF as lesion sites to evaluate the cholinergic interventions observed in aging and cognition. Studies with selective BF neurotoxic lesion concluded this region was associated with cognitive function characterized by attention (Muir et al., 1993; Baxter et al., 1997, 1999; Bucci et al., 1998; Chiba et al., 1999; Chudasama et al., 2004), and learning and memory (Hepler et al., 1985; Hagan et al., 1988; Berger-Sweeney et al., 1994; Baxter et al., 1995; Janisiewicz et al., 2004). Furthermore, a significant correlation between cognitive impairment and decline in cholinergic markers for the septo-hippocampal projection in aged rats (Gallagher et al., 1990; Smith and Booze, 1995) supports the involvement of BF cholinergic neurons in cognition. More recently, animals with a saporin-induced partial loss of septo-hippocampal cholinergic neurons exhibited cognitive deficit (Brayda-Bruno et al., 2013). Other lesion studies extended the importance of the BF cholinergic system in the process of functional recovery from brain injury in young rats (Conner et al., 2005), which is intriguing in that animals with BF lesions may show deficits in cognitive function. Selective lesion of cholinergic inputs to the hippocampus has also been used to evaluate the effects of cholinergic receptors in regulating hippocampal ACh release (Thorne and Potter, 1995).

Though lesion studies have generated substantial controversy, the advent of this approach has generated new empirical tests and compelling information on the potential function of cholinergic and non-cholinergic neurons emanating from the BF. Additionally, this has unveiled the possible role played by loss of these neurons in stress, aging, and other neurocognitive diseases. More essentially, animal models are indispensable for recreating specific human pathogenic events, and invaluable for drug screening and therapeutic intervention assessment.

## HPA Axis and Glucocorticoids in Stress Regulation and Aging

**Figure 2:**
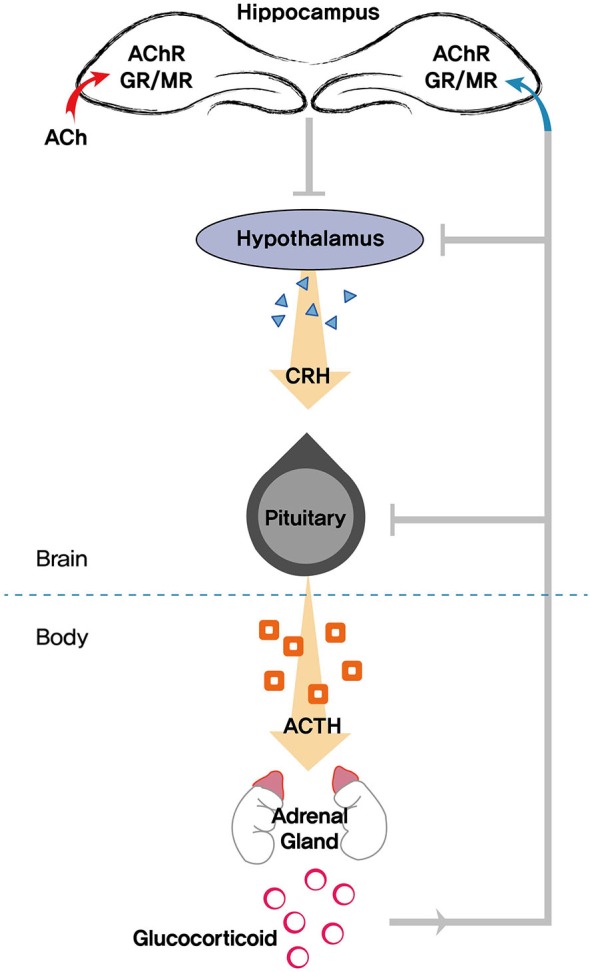
**Schematic diagram showing the role of the hippocampus in modulating the HPA (hypothalamic pituitary adrenocortical) axis**. Stress triggers the release of glucocorticoids, which exerts feedback to the hippocampus, hypothalamus, and pituitary. Acetylcholine (ACh) is also involved in mediating neuroendocrine, emotional, and physiological responses in tandem with the HPA axis. AChR, acetylcholine receptors; GR, glucocorticoid receptor; MR, mineralocorticoid receptor; CRH, corticotropin-releasing hormone; ACTH, adrenocorticotropic hormone.

The HPA axis is an integral component in promoting resilience to stress. Most salient age-related changes in the stress response are thought to occur in the HPA axis. The HPA system is classically controlled by a range of afferent signals responsible for the coordinated release of various signaling markers. The hypothalamus is at the top of the hierarchy in the control of the central HPA axis. When the brain perceives a stressor, activation of the paraventricular nucleus of the hypothalamus triggers the release of corticotropin-releasing hormone. Corticotropin-releasing hormone stimulates pituitary adrenocorticotropic hormone (ACTH) release and activates the adrenal gland, which secretes glucocorticoids (cortisol in humans and corticosterone in rodents). Glucocorticoids released from the adrenal gland interact with the HPA axis by binding to specific GRs in the brain, forming a closed-loop feedback system and subsequently coping with stress (Sapolsky et al., 1986; Mizuno and Kimura, 1997; Srinivasan et al., 2013). Thus, glucocorticoids are the end product of the HPA axis and regulate a wide array of actions influencing neuronal function and metabolism (Figure 2).

Two types of steroid hormone receptors have been identified in tandem in the brain: the mineralocorticoid receptor (MR) and the GR. Expression of MR is considerably more restricted in the brain compared to the ubiquitous expression of GR, and both mediate classical genomic and non-genomic glucocorticoid actions by acting as nuclear transcriptional activators and repressors (Reul and de Kloet, 1985; Joëls, 2008; van der Laan and Meijer, 2008; Groeneweg et al., 2011). Moreover, the secretion of glucocorticoids and their binding to receptors leads to auto-regulatory decreases in receptor availability and *vice versa* (Sapolsky et al., 1984; Reul et al., 1987a,b). Actions of GR described above extends the role of GR beyond mediating glucocorticoid feedback following stress, whereas MR participates in basal HPA tone (Reul et al., 1987a; De Kloet et al., 1998) and the genomic action in adaptation and homeostasis after stress exposure (de Kloet, 2008). After GR binding, these activated receptors are translocated to the nucleus where they bind to the glucocorticoid responsive elements (GRE) and affect the transcriptional activity of target genes (Funder, 1997). Stress causes a down-regulation of GR through the increase of circulating glucocorticoids, eventually decreasing sensitivity to nuclear transcriptional activities. Glucocorticoid-GR can also activate transcription by binding directly as a homodimer to the GRE DNA sequence present in the promotors of target genes (e.g., *serum- and glucocorticoid-induced protein kinase, mitogen-activated protein kinase phosphatase-1*, etc) that has been reported to regulate cognitive function (Lee et al., 2007; Vyas and Maatouk, 2013; Cestari et al., 2014).

HPA activity tends to increase with age due to inefficiencies of glucocorticoid negative-feedback inhibition, resulting in elevated plasma levels of ACTH and glucocorticoids (Mizoguchi et al., 2009; Aguilera, 2011). A decrease in glucocorticoid negative-feedback inhibition is associated with the loss of GR and altered GR signaling in the forebrain (Issa et al., 1990; Bizon et al., 2001; Lund et al., 2004; Lee et al., 2012). Increased levels of glucocorticoids appear to threaten hippocampal neurons and direct the loss of dendrite complexity in the hippocampus (Hibberd et al., 2000). Inappropriate HPA axis regulation in aging is reviewed by Aguilera (2011) who stresses its adverse effect in stress-related brain disorders.

A link between HPA deregulation and disruption of GR has been observed in AD and other neurodegenerative diseases (Brureau et al., 2013; Vyas and Maatouk, 2013). Interestingly, though extra-hypothalamic GR sites receive attention for this glucocorticoid-mediated HPA inhibitory feedback; the hippocampus receives the utmost attention with regards to these effects (McEwen et al., 1968, 1969; Sapolsky et al., 1984, 1986; Herman et al., 1989). The influence of glucocorticoids on the HPA axis markedly depends on the available GR in individual tissues (Simons, 2008). Abundant expression of GR in the forebrain (McEwen et al., 1969; McEwen and Wallach, 1973), and increased activity of the HPA axis in the absence of forebrain inhibition on HPA axis by damage of the forebrain (Jacobson and Sapolsky, 1991; van Haarst et al., 1996), contributes to the sequelae associated with GR malfunction. Taken together, forebrain GR expression is critical for HPA axis regulation in response to stress (Furay et al., 2008) and further adduces the role of GR in stress.

## Cholinergic Neuron in Stress Regulation via HPA Axis and Cognitive Function

A further increase in ACh release was observed in the hippocampus after acute stress (Finkelstein et al., 1985; Gilad et al., 1985; Imperato et al., 1989). Corticosterone administration, mimicking the increase in the plasma corticosterone concentration produced by stress, induced hippocampal ACh (Imperato et al., 1989). These studies support the involvement of glucocorticoids in the cholinergic innervation of the hippocampus, and the activation of the HPA axis in the process. Stress-induced responses activated the septo-hippocampal cholinergic pathway within minutes (Gilad, 1987), which then induced ACh-mediated neuroendocrine, emotional, and physiological responses by stimulating the HPA axis (Newman et al., 2001). This HPA axis activation led to the release of corticosterone, a stress neurohormone (Nyakas et al., 1987; Calogero et al., 1988, 1990). Increased release of hippocampal ACh and glucocorticoids in response to stress was observed in young rats, but not in aged rats (Mizuno and Kimura, 1997). However, contradictory results have been reported. Proteomic analyses of the hippocampus of rats exposed to stress showed a decrease in a precursor protein of hippocampal cholinergic neurostimulating peptide (HCNP) leading to a loss of ACh production (Kim and Kim, 2007). HCNP stimulate the enzyme activity of choline acetyltransferase in neurons. It is also reported that expression levels of HCNP precursor protein mRNA were decreased in the hippocampus of AD patients (Maki et al., 2002). These reports indicate a possible intricate interplay between the level of glucocorticoids, ACh, and hippocampal cholinergic protein expression effecting septo-hippocampal cholinergic pathways.

Interestingly, a parallel increase in both plasma corticosterone and hippocampal ACh level has been validated as a consequence of elevated platform exposure, a relatively mild stress (Degroot et al., 2004). The interaction of glucocorticoid with ACh in the brain is well reviewed (Mora et al., 2012). Moreover, regulation of the HPA axis by glucocorticoid feedback and cholinergic brain function modulating stress responses depends on the intensity and predictability of stressful stimuli (Pitman et al., 1988; Martí and Armario, 1997; Morris and Rao, 2014).

Although ACh mediates its effects via both types of ACh receptors, mAChR are more involved in cognitive impairment and are densely present in the hippocampus (Dutar et al., 1995; Colgin et al., 2003; Drever et al., 2011). An excessive loss of mAChR in the hippocampus of Alzheimer’s patients, and severely impaired muscarinic signaling associated with age-related cognitive decline (Bartus et al., 1982; Zhang et al., 2007), reveal the connection between the muscarinic-dependent cholinergic system and cognitive impairment. Scopolamine-induced mAChR blockade resulted in cognitive deficit in healthy adult humans (Voss et al., 2010). Additionally, mAChR antagonists significantly elevated plasma corticosterone in stressed rats, suggesting an inhibitory effect of mAChR stimulation on pituitary-adrenal function (Kile and Turner, 1985). This result is indicative of a correlation between corticosterone levels, mAChR availability, and cognitive function.

### Basal Forebrain Cholinergic Neurons, Hippocampal Glucocorticoids, and Glucocorticoid Receptor in Stress Regulation and Cognitive Aging

The hippocampus is a brain structure crucially involved in memory, the neuroendocrine regulation of stress hormones, and termination of the stress response via HPA axis glucocorticoid-mediated inhibition (Mizuno and Kimura, 1997; Kim and Diamond, 2002). Studies showing hippocampal damage due to prolonged exposure to glucocorticoids or chronic stress in primates (Uno et al., 1989; Sapolsky et al., 1990) have been triggered an assessment of the cumulative impact of such exposures on the hippocampus using other animals. A number of studies reported that chronic stress or glucocorticoids contributed hippocampal cell death in adult rats (Sapolsky et al., 1985; Dachir et al., 1997). The hippocampus contains a high density of GR and is a target of glucocorticoid actions. Cognitive deficits are associated with a loss of hippocampal neurons, in particular pyramidal cells, due to increased glucocorticoid exposure (McEwen et al., 1968; McEwen, 1999; Hibberd et al., 2000). However, others failed to find hippocampal neuronal loss in rats (Bodnoff et al., 1995; Sousa et al., 1998; Coburn-Litvak et al., 2004), tree shrews (Vollmann-Honsdorf et al., 1997; Fuchs et al., 2001), primates (Leverenz et al., 1999), and humans (Müller et al., 2001). The inconsistencies in literatures on glucocorticoid-related cell death may arise from species-specific differences in expression levels of GR and MR (Conrad, 2008).

In any case, impairments of hippocampal dependent memory and synaptic plasticity, and structural alterations have been observed in the animals with chronic stress or corticosterone treatment (Bodnoff et al., 1995; Fuchs et al., 2001; Finsterwald and Alberini, 2014). Down-regulation of GR in the hippocampus follows the chronic corticosterone treatment. (Tornello et al., 1982). And GR signaling was altered in stress-related psychopathologies (Finsterwald and Alberini, 2014). Thus this indicates a connection between GR availability and cognitive function. As GR are responsible for negative feedback control of the adaptive stress response (De Kloet et al., 1998), a reduction in hippocampal GR is associated with post-stress glucocorticoid hypersecretion (Sapolsky, 1996). Additionally, glucocorticoid treatments exacerbated the cholinergic neurotoxin ethycholine aziridinium (AF64A)-induced cholinergic lesions in the hippocampus, suggesting a pathophysiological link between glucocorticoids and age-dependent declines in cholinergic function or cholinergic degeneration in AD (Hörtnagl et al., 1993). Moreover, the essential coordinating role of GR in regulating glucocorticoid secretion through the HPA axis in response to stress in aging is well documented (Issa et al., 1990; Herman et al., 1996; Bizon et al., 2001; Murphy et al., 2002; Furay et al., 2008; Mizoguchi et al., 2009).

Glucocorticoid receptor, when functioning as a ligand-dependent transcription factor, controls transcription by directly binding to positive and negative GRE, regulating transcriptional increases (anti-inflammatory) or decreases (HPA axis negative feedback) or inhibiting transcriptional activity of other factors (on pro-inflammatory molecules) (Silverman and Sternberg, 2012). The GR-DNA binding phenomenon is gradually receiving recognition, and research on the anti-inflammatory effects of GR-DNA binding has been reported in *in vivo* and *in vitro* studies (Reichardt et al., 1998, 2001; Schäcke et al., 2002; Clark, 2007). Target disruption of GR genes and impaired GR-DNA binding has been correlated with cognitive deficits in mice (Oitzl et al., 1997, 2001), while intra-hippocampal GR blockade with a GR antagonist produced memory impairments (Nikzad et al., 2011). Diminished GR signaling and GR mRNA in the aged hippocampus is related to memory impairment and HPA axis dysregulation (Bizon et al., 2001; Murphy et al., 2002; Lee et al., 2012). Furthermore, a decrease in the nuclear uptake of corticosterone, decreased nuclear translocation, and DNA binding deficits were observed in the hippocampus of the aged rat (Sapolsky et al., 1983; Murphy et al., 2002; Lee et al., 2012). This highlights the need to understand glucocorticoids-genomic interactions, as this may illuminate the role of GR in cognitive processes. Reduced expression of GR mRNA in the hippocampus and medial prefrontal cortex was also observed with memory-impaired aged rats relative to young controls and memory-unimpaired aged rats, with no change in the basal levels of circulating glucocorticoids (Bizon et al., 2001). Another source of GR signaling interference in hippocampal cognition may be mediated by the regulation of other intruding nuclear transcriptional factors, such as activator protein and nuclear factor κB (NF-κB; Yang-Yen et al., 1990; McKay and Cidlowski, 1998; Lund et al., 2004). Recently, reduced expression of FKBP5, a key GR modulator, and smaller hippocampal volumes were observed in posttraumatic stress disorder, which was reversed after cognitive behavioral therapy (Levy-Gigi et al., 2013). Supporting the above facts, decreased nuclear GR mRNA and protein was observed in aged rats with cognitive impairment, suggesting defective GR transport might affect the transcriptional properties of hippocampal neurons with HPA axis dysfunction and could have age-related impact on cognitive decline and the loss of stress regulation (Bizon et al., 2001; Lee et al., 2012).

Although high levels of glucocorticoids are not associated with the loss of hippocampal neurons (Leverenz et al., 1999), some studies suggest interplay between the accumulative factor of stress and aging in the process of cell loss (Hibberd et al., 2000). On the other hand, Notarianni recently proposed a role for GR signaling in the initiation and development of AD, implicating over-activation of GR with hypercortisolemia in promoting amyloid beta (Aβ) production that leads to Aβ deposition and associated neuroinflammation (Notarianni, 2013).

Chronic neuroinflammation in the BF is also linked to loss of cholinergic neurons and is responsible for cognitive impairment associated with aging and AD (Willard et al., 1999). Significant loss of cholinergic neurons in the MS/diagonal band was also observed in aged animals with memory impairment (Baskerville et al., 2006). A direct correlation between glucocorticoid regulation of GR via the HPA axis and impaired GR function as a mechanism for inflammation is well reviewed (Silverman and Sternberg, 2012), emphasizing its importance in the prevention and management of chronic stress. Further, upregulation of pro-inflammatory cytokines occurs in the cortex and hippocampus of rats with post-surgery stress, resulting in post-operative cognitive dysfunction. This surgery-induced inflammation can be reduced by acetylcholinesterase inhibitors (Kalb et al., 2013), pointing to the involvement of the cholinergic system in cognitive impairment associated with neuroinflammation.

### Interaction of ACh Receptor and GR in the Hippocampus

Cognitive impairment was observed in healthy human subjects treated with scopolamine, a selective mAChR antagonist (Voss et al., 2010). Rats treated with scopolamine showed spatial working memory impairment in an 8-arm radial maze task and alterations in ventral hippocampi ACh release (Mishima et al., 2000). In addition, impairment of recognition memory in BF cholinergic lesioned animals was aggravated by scopolamine, emphasizing the importance of mAChR in cognitive function (Steckler et al., 1995). Deficit in the transduction of cholinergic mAChR signals has been detected in the hippocampus of aged rats (Smith and Booze, 1995), the cortex of aged monkeys (Vannucchi and Goldman-Rakic, 1991), and in AD patients (Flynn et al., 1991). Additionally, impaired mAChR binding was found in the striatum and hippocampus of aged rats (Anson et al., 1992; Yamagami et al., 1992; Nieves-Martinez et al., 2012).

Loss of mAChR exacerbates cognitive decline and AD pathology, such as increased plaques/tangles and cerebrovascular deposition of Aβ in AD mice (Medeiros et al., 2011). Treatment with selective muscarinic agonists resulted in reduced production of Aβ in AD patients (Hock et al., 2003). A speculative pathway, due to loss of mAChR function in rats, induced by selective hippocampal cholinergic lesions with AF64A is predicted to influence effects in stimulating nicotinic receptors that may modulate the release of ACh (Thorne and Potter, 1995). In context, it is predicted that loss of mAChR function might exert stimulatory effects on nicotinic receptors, which are well described in cognitive functions (Levin, 2013). This highlights the importance of mAChR in BF cognitive function. Decreased expression of mAChR in the hippocampal CA1 region of aged epileptic animals (Cavarsan et al., 2011), and severely impaired muscarinic signaling in the hippocampus of cognitively impaired rats (Zhang et al., 2007), illustrates the involvement of the cholinergic system with cognition. More recently, decreases in ACh and mAChR were observed in cognitively impaired mice (Park et al., 2013). Additionally, impaired hippocampal ACh release and cognitive deficits in mAChR knockout mice (Tzavara et al., 2003) coupled with mAChR antagonist impairment of memory in aged rats (Quirion et al., 1995; Klinkenberg and Blokland, 2010) implies a role for mAChR in the cholinergic hypothesis of cognition.

Activation of septo-hippocampal cholinergic neurons is manifested by increased release of ACh and choline uptake. This choline uptake is reduced below control levels in the presence of chronic stress, followed by an up-regulation of muscarinic binding sites (Finkelstein et al., 1985). Suppression of glucocorticoid secretion enhances hippocampal cholinergic transmission in rats (Mizoguchi et al., 2008). Furthermore, enhanced memory consolidation by striatal corticosterone injection was blocked by administration of scopolamine (Sánchez-Resendis et al., 2012), confirming the interactive relationship between glucocorticoids and cholinergic receptor. Glucocorticoid modulation of mAChR in lung (Scherrer et al., 1997), smooth muscle (Emala et al., 1997), chronic obstructive pulmonary disease (Johnson, 2005), and several brain nuclei in rats (Torres et al., 1991) suggests a similar pattern in hippocampal cholinergic neurons.

Rats with selective removal of hippocampal cholinergic input showed HPA axis dysfunction and decreased hippocampal GR levels (Han et al., 2002; Helm et al., 2002, 2004; Lim et al., 2012). Subsequent studies also revealed altered GR-protein kinase A (PKA)-NF-κB signaling in the hippocampus with loss of cholinergic input (Lim et al., 2011, 2012). The study regarding interactive effects of stress with loss of BFCN on cognitive function reported chronic stress induced impairment of working memory in rats with loss of hippocampal cholinergic input (Craig et al., 2008). Recently, we examined whether chronic stress aggravated cognitive deficit induced by selective BF cholinergic lesions leading to alterations in GR-PKA-NF-κB signaling. Lesioned rats receiving chronic stress showed a severe impairment in spatial memory and increased NF-κB signaling activation, which was substantiated by increased hippocampal pro-inflammatory gene expression, such as inducible nitric oxide synthase and cyclooxygenase-2 (Lee et al., 2013). These data indicate that the interaction between GR and ACh receptors is associated with stress-induced cognitive dysfunction.

## Conclusion

The present review summarizes the current research and shows the importance of glucocorticoids and their receptor in modulating cognition in stress, aging, and AD via the cholinergic system. This may pave a new way in understanding the progression of the aforementioned diseases. Therefore, this review facilitates the understanding of the following: (1) involvement of both GR and ACh receptors in modulating cognition, thus providing a palliative approach for pharmaceutical interventions as these receptors are discussed in many research papers as a route to therapeutic intervention; (2) an interactive platform to mark the holistic consequences of cognitive impairment converging from varied neurological deficits; and finally; (3) an interactive role of glucocorticoids in the development of cognitive dysfunction and vulnerability of the hippocampus to such exposure. The main objective of this review was not only to highlight the possible underlying association between the various pathways and neural circuits involved in cognitive impairment, but also to enable the mitigation of such stress-induced cognitive morbidity by developing more effective pharmacotherapeutic strategies to ameliorate such diseases.

## Conflict of Interest Statement

The authors declare that the research was conducted in the absence of any commercial or financial relationships that could be construed as a potential conflict of interest.
